# Fragility Fracture Network Position on Unrestricted Weight-Bearing After Hip Fracture Surgery

**DOI:** 10.1177/21514593251351136

**Published:** 2025-07-01

**Authors:** Ruqayyah Turabi, Frede Frihagen, Rhona McGlasson, David Wyatt, Alex Trompeter, Lauren Beaupre, Luiz Fernando Cocco, Matthew Costa, José Luis Dinamarca-Montecinos, Juan Carlos Viveros-García, Jae-Young Lim, Joon-Kiong Lee, Hui Min Khor, Cristina Ojeda-Thies, Monica Perracini, Takeshi Sawaguchi, Julie Switzer, Irewin Tabu, Ronald Man Yeung Wong, Wei Mao, Katie Jane Sheehan

**Affiliations:** 1 Department of Population Health Sciences, School of Life Course and Population Sciences, 4616 King’s College London, UK; 2 Department of Physical Therapy, College of Nursing and Health Sciences, Jazan University, Saudi Arabia; 3 Department of Orthopaedic Surgery, 60517 Østfold Hospital Trust, Grålum, Norway; 4 Institute of Clinical Medicine, University of Oslo, Norway; 5 Fragility Fracture Network; 6 City St George’s, University of London, St George’s University Hospital, UK; 7 Department of Physical Therapy, Faculty of Rehabilitation Medicine, 3158 University of Alberta, Edmonton, Canada; 8 Fragility Fracture Network Brasil (FFN Brazil), Department of Orthopedics and Traumatology, 58804 Escola Paulista de Medicina, Federal University of São Paulo, Brazil; 9 Nuffield Department of Orthopaedics, Rheumatology and Musculoskeletal Sciences, 6396 University of Oxford, UK; 10 Facultad de Medicina, 28068 Universidad de Valparaíso, Chile; 11 Hospital Regional ISSSTE Léon, Guanajuato, Mexico; 12 Department of Rehabilitation Medicine, 26725 Seoul National University Bundang Hospital, Seoul National University College of Medicine, Seoul, Republic of Korea; 13 Department of Orthopaedic Surgery, Beacon Hospital, Petaling Jaya, Malaysia; 14 Department of Medicine, Faculty of Medicine, 37447 University of Malaya, Kuala Lumpur, Malaysia; 15 Department of Traumatology and Orthopedic Surgery, 542561 Hospital Universitario 12 de Octubre, Madrid, Spain; 16 Department of Surgery, School of Medicine, Complutense University of Madrid, Spain; 17 Programa em Fisioterapia, 149944 Universidade Cidade de São Paulo, Brazil; 18 Department of Traumatology, Fukushima Medical University, Japan; 19 Department of Orthopaedic Surgery, University of Minnesota Twin Cities, Minneapolis, USA; 20 University of the Philippines Manila, Manila, Philippines; 21 Department of Orthopaedics & Traumatology, 26451 The Chinese University of Hong Kong, China; 22 Department of Orthopaedics, Shanghai Sixth People’s Hospital, Shanghai Jiao Tong University, China; 23 Bone and Joint Health, 12476 Blizard Institute, Queen Mary University of London, UK

**Keywords:** hip fracture, rehabilitation, orthogeriatric care, weight-bearing, international collaboration

## Abstract

**Objectives:**

This position paper from the Fragility Fracture Network (FFN) responds to the observed global variation in weight-bearing prescriptions after hip fracture surgery in older adults.

**Methods:**

The paper summarises current guidelines and evidence regarding unrestricted weight-bearing after hip fracture surgery.

**Results:**

The synthesis of available evidence supports the endorsement of unrestricted weight-bearing after surgery to enhance patient outcomes.

**Conclusion:**

The FFN endorses unrestricted weight-bearing and recommends healthcare professionals, institutions, and policymakers re-evaluate practices favouring limited or non-weight-bearing prescriptions and establish a standardised system for monitoring and auditing, with clear justification and documentation of any restrictions.

## Introduction

Hip fractures are associated with persistent pain, disability, and increased mortality.^1^ Early surgery is the treatment of choice with a primary objective to alleviate pain and restore pre-fracture mobility.^2,3^ To facilitate this, unrestricted weight-bearing after hip fracture surgery is recommended by national guidelines.^4,5^

**Figure 1.**
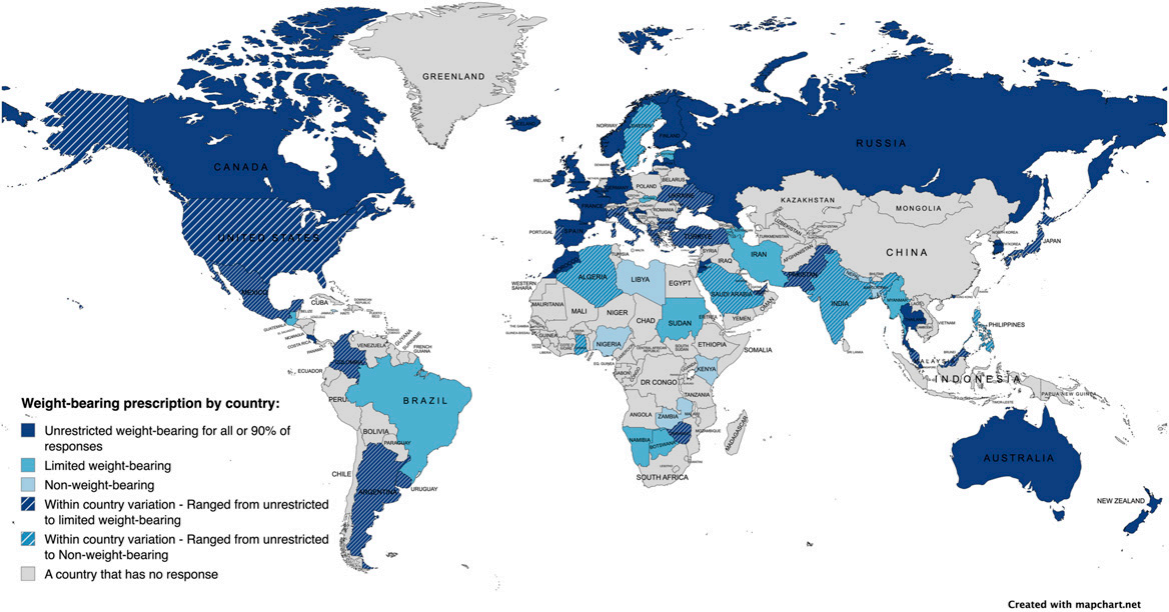
Response to ‘Most Frequent Weight-Bearing Prescription’ by Country^6^

Despite these recommendations, a recent survey of 389 health professionals from across 71 countries reported considerable global variation in weight-bearing prescription after hip fracture surgery.^6^ Overall, 73.5% of healthcare professionals reported unrestricted weight-bearing as the prescription of choice, with a notable disparity between high-income countries (86.3%) and low- and middle-income countries (41%) (Figure 1).

The Fragility Fracture Network (FFN) is an international organisation that focuses on enhancing the multidisciplinary care of patients with fragility fractures, including efforts for secondary prevention (https://fragilityfracturenetwork.org/).

This FFN position paper serves as a response to the observed global variation in weight-bearing status as part of the patient’s rehabilitation prescription. The FFN endorses unrestricted weight-bearing after hip fracture surgery. This paper aims to substantiate this endorsement by the following means:

1. A summary of current guidelines on weight-bearing after hip fracture surgery.
2. A summary of the evidence on the association between unrestricted weight-bearing and outcomes.

Further, this paper will report barriers and facilitators to unrestricted weight-bearing to aid healthcare professionals, institutions, and policymakers in implementing this best practice.

## Guidelines Summary

For the purpose of this position paper, recently recommended terminology to define weight-bearing will be employed: non weight-bearing, limited weight-bearing, or unrestricted weight-bearing.^7^ This terminology represents the only validated, consensus-based standard, addressing the variability in weight-bearing terminology.^8^

Table 1 presents a summary of guidelines and their weight-bearing statements (retrieved from websites, and/or following a request from FFN to its membership). The National Institute for Health and Care Excellence, American Academy of Orthopaedic Surgeons, British Orthopaedic Association, Canadian guidelines, Australian and New Zealand guidelines, Spanish guidelines and Norwegian guidelines all support unrestricted weight-bearing postoperatively. Malaysia and South Korea support unrestricted weight-bearing after arthroplasty, with team collaboration determining its timing and level based on the type of hip fracture and surgical approach. Of note, the Canadian and Swedish guidelines do not specify restrictions. Brazil cites The National Institute for Health and Care Excellence, the American Academy of Orthopaedic Surgeons, however, does not specify weight-bearing restrictions.

**Table 1.** Guidelines Weight-Bearing Statements

Country	Guidelines/ source	Weight-bearing statement from the guidelines^a^
Australia and New Zealand	The Australian and New Zealand Guideline^9^	“Operate on patients with the aim to allow them to fully weight bear (without restriction) in the immediate post-operative period.”
		“The committee considered that the recommendation around unrestricted weight bearing post-operatively is appropriate and no modifications are required for the Australian and New Zealand context.”
Brazil	Brazilian guidelines for fracture treatment of the femoral neck in older adults^10^	“Question 14: For older adults patients with femoral neck fractures undergoing surgical treatment with osteosynthesis, partial or total arthroplasty, does early mobilization and weight-bearing with assistance accelerate recovery?
		The American Academy of orthopaedic surgeons: The studies showed functional improvement, leg strengthening, balance, mobility and improvement in activities of daily living at home. Evidence: Moderate
		The national institute for health and care excellence (NICE): NICE recommends that daily physiotherapy has potential benefits in improving mobility and balance, increasing independence and reducing the need for institutional and social care.”
Canada	Canadian guidelines/ position paper.^11-13^	“Weight bearing as tolerated and activity as tolerated within 24 hours following surgery”
		“Weight bearing as tolerated and activity as tolerated, no activity restrictions for hemi arthroplasty and fixations unless specified by surgeon”
		“Encourage patient to weight-bear, as tolerated, unless otherwise indicated. For patients who have been previously mobile, the need for immediate weight-bearing as tolerated is of paramount importance in promoting future recovery as it has been shown to decrease medical complications, decrease mortality, and improve functional recovery and functional outcomes.”
		“Given the negative consequences and ineffectiveness of weight-bearing restrictions in hip fracture patients, they should be avoided, and the vast majority of patients should be allowed to mobilize and weight bear as tolerated. Weight-bearing restrictions may be warranted in younger patients who undergo fixation of intra-capsular hip fractures in attempts to salvage the native joint. In these rare situations in which restricted weight-bearing is warranted, a clear plan for progression of mobility and weight bearing should be in place prior to hospital discharge”
Malaysia	Ministry of health Malaysia^14^	“Arthroplasty is the preferred choice in non-displaced fracture neck of femur in geriatric patients for early full weight-bearing ambulation.”
		“Communication between multidisciplinary team members is crucial to determine suitable timing and level of weight bearing which will depend on the aspects of hip fracture, types of hip surgery and findings at the time of surgery.”
Norway	Norwegian guidelines/ papers.^15,16^	“Osteosyntheses and prostheses are load-stable immediately postoperatively, and the patient should be mobilized without restrictions. Early and full mobilization is the rule; in case of exception, this should be well documented. Early mobilization, preferably already the day of surgery, and guided by a physiotherapist, is sought”
		“Early mobilization with weight-bearing exercise programs and participation in activities of daily living should be executed by both physical therapists and nursing staff.”
		“Early mobilization with weight-bearing activities after a hip surgical procedure is highly recommended and should be initiated within the first postoperative day”
		“This patient group is usually unable to partially weight-bear in a controlled manner”
South Korea	South Korean clinical practice Guidelines^17^	“Operate on patients with the aim to allow them to fully weight bear (without restriction) in the immediate post- operative period.”
		“Weight-bearing on the injured leg should be allowed, unless there is concern about quality of the hip fracture repair (eg, poor bone stock or comminuted fracture).”
		“We suggest that weight-bearing exercise is recommended after HFS, but close communication between surgeons and rehabilitation physicians is required to determine the timing and level of weight-bearing exercises.”
Spain	Clinical practice guidelines on the care of the older patient with hip Fracture^18^	“Early mobilization can prevent medical complications such as pressure ulcers, deep vein thrombosis and mortality so weight bearing should be performed as soon as possible.”
		“Once the patient is standing, balance will be worked on and walking will be re-educated, allowing weight bearing, as tolerated, except for those patients in whom it is contraindicated due to medical or surgical complications (grade of recommendation A).”
Sweden	National hip fracture care Program^19^	“Early mobilization and loading of the injured leg reduces the risk of complications. Early mobilization is equally important every day of the week and therefore all staff groups must be involved in this.”
		On arthroplasty “movement restrictions, to reduce the risk of dislocation, probably has no effect but has not been studied in posterior incisions”
United Kingdom	The National Institute for Health and Care Excellence^4^	“Operate on people with the aim to allow them to fully weight bear (without restriction) in the immediate postoperative period.”
United Kingdom	The British Orthopaedic Association (BOA)^20^	“All surgery in the frail patient should be performed to allow full weight-bearing for activities required for daily living and within 36 hours of admission, in line with current hip fracture care. Patients should be seen by a physiotherapist on postoperative day 1 with early identification of functional rehabilitation goals as detailed in the rehabilitation BOAST.”
United States	The American Academy of orthopaedic Surgeons^5^	“Following surgical treatment of hip fractures, immediate, full weight bearing to tolerance may be considered.”

^a^Several guidelines contain identical or similar weight-bearing statements. To maintain conformability, Table 1 presents these statements as they appear in the original sources.

## Weight-Bearing and Outcomes

Unrestricted weight-bearing is associated with improved functional outcomes, including reduced postoperative pain and increased mobility,^21-23^ as well as a higher likelihood of being discharged to home vs to rehabilitation or nursing facilities.^22,24,25^ In contrast, non- or limited- weight-bearing is associated with loss of mobility, which can adversely affect overall recovery.^24^

Unrestricted weight-bearing from the first postoperative day results in fewer major and minor complications, such as deep vein thrombosis, pulmonary embolism, urinary tract infections, pressure sores, delirium, transfusion, and mortality within the first 30 days after surgery.^25,26^ Additionally, there is no evidence to suggest an association between unrestricted weight-bearing and the risk of revision surgeries due to fixation failure.^21,27^ On the other hand, non- or limited- weight-bearing is associated with a higher incidence of adverse events, including increased mortality, surgical site infections, pneumonia, cardiac arrest, delirium, and deep vein thrombosis.^26,28^ Notably, a study by Ottesen and colleagues,^26^ which controlled for factors such as demographics, comorbidities, functional level and procedure type, found that patients prescribed limited weight-bearing were nearly 60% more likely to die within 30 days compared to those prescribed unrestricted weight-bearing.

Unrestricted weight-bearing is associated with shorter hospital stays,^23,25^ reducing overall hospital costs. In contrast, limited weight-bearing is associated with increased hospital stay.^26,29^ Unrestricted weight-bearing is also associated with a greater likelihood of home discharge compared to limited- or non- weight-bearing, reducing cost and burden on social care systems.^30^

## Implementation Challenges

Despite these recommendations, non- or limited- weight-bearing protocols are still in place. The rationale behind limited/non weight-bearing prescriptions includes patient-related factors such as poor bone quality from advanced osteoporosis and the fracture type (ie, subtrochanteric fracture), process-related factors such as the surgery type, reduction achieved, or the risk of implant failure, and structure-related factors such as the surgeries completed before holiday periods.^30-35^

In addition, clinicians identified further challenges in clinical practice and healthcare systems. Variability in training, the absence of standardised protocols, reliance on subjective experience over evidence-based guidelines, and the lack of routine audits and evaluations collectively lead to inconsistencies in practice.^31,35^ These challenges underscore the need for evidence-informed standardisation to optimise care.

Modern implants support unrestricted weight-bearing when fracture reduction is adequate, and the implant is appropriately positioned. Surgeons tend to accept that arthroplasty removes concerns about fracture healing altogether. Intramedullary fixations offer load-sharing, allowing for weight-bearing, and extramedullary implants, such as sliding hip screws, can be used successfully under unrestricted weight-bearing protocols, provided surgeons achieve adequate reduction and secure fixation.^36^

Despite this, inconsistencies in weight-bearing protocols persist, with allowances for non- or limited weight-bearing often justified by implant type or perceived fracture stability. Such justifications may reflect clinical caution or health system limitations rather than absolute contraindications, especially when evidence suggests that unrestricted weight-bearing does not increase fixation failure.^31,37-39^ Importantly, older adults with hip fractures often struggle to comply with weight-bearing restrictions, tending to load the limb as needed to mobilise.^39,40^ This may lead some clinicians to adopt a precautionary approach, selectively applying restrictions to those they believe are more likely to adhere to the restriction, further contributing to variation in practice.

Furthermore, many guidelines support unrestricted weight-bearing, and some include conditional phrasing such as “may be considered” or “unless otherwise indicated”. This language allows for clinical discretion in complex cases, but it may also contribute to variation in implementation by permitting more conservative interpretations. Recent consensus acknowledges that in cases where non or limited weight-bearing is prescribed, it should be explicitly justified, with a clear rationale, defined duration, and specific nature of the restriction.^7^

## Recommendation

This paper represents the global FFN position that advocates for the prescription of unrestricted weight-bearing following hip fracture surgery in response to observed global variations in clinical practice. This endorsement is supported by the available evidence indicating that unrestricted weight-bearing improves clinical outcomes.

The FFN also recommends that healthcare professionals, institutions, and policymakers worldwide re-evaluate practices which lead to the prescription of limited- or non- weight-bearing prescriptions after hip fracture surgery in their organisations, in the face of increasing and compelling evidence demonstrating the benefits of unrestricted weight-bearing protocols.

We advocate for a standardised system to monitor and audit weight-bearing status, ensuring that if limited- or non- weight-bearing is prescribed, the duration of the restriction and rationale are clearly documented. The FFN serves to actively promote the standardisation of orthogeriatric care following hip fracture, inclusive of unrestricted weight-bearing protocols. This is achieved through advocacy, education, and collaboration with professional organisations from both FFN Global and the network of National FFNs. Several countries have already integrated unrestricted weight-bearing into their national hip fracture guidelines, and further collaboration is needed to support global adoption.

There should be global consistency in applying evidence-based protocols to ensure that all patients benefit equally from the latest best practices in hip fracture management. By incorporating regular audits and monitoring into routine practice, adherence to best practices such as unrestricted weight-bearing can be promoted, facilitating continuous improvement in patient care.

## ORCID iDs

Ruqayyah Turabi https://orcid.org/0000-0001-6439-6276

Frede Frihagen https://orcid.org/0000-0002-4811-669X

David Wyatt https://orcid.org/0000-0001-5859-7389

Alex Trompeter https://orcid.org/0000-0001-7887-497X

José Luis Dinamarca-Montecinos https://orcid.org/0000-0002-0186-5992

Jae-Young Lim https://orcid.org/0000-0002-9454-0344

Cristina Ojeda-Thies https://orcid.org/0000-0001-7052-1491

Julie Switzer https://orcid.org/0000-0002-4834-1643

Irewin Tabu https://orcid.org/0000-0001-5952-128X

Katie Jane Sheehan https://orcid.org/0000-0002-5325-7454
